# Effects of oral targeted treatments in pulmonary arterial hypertension: A systematic review and meta-analysis

**DOI:** 10.3389/fcvm.2022.915470

**Published:** 2022-08-02

**Authors:** Hui-ru Zhu, Hong-yu Kuang, Qiang Li, Xiao-juan Ji

**Affiliations:** ^1^National Clinical Research Center for Child Health and Disorders, Department of Ultrasound, Children’s Hospital of Chongqing Medical University, Chongqing, China; ^2^Chongqing Key Laboratory of Pediatrics, Ministry of Education Key Laboratory of Child Development and Disorders, Chongqing, China; ^3^Department of Cardiology, The Second Affiliated Hospital of Chongqing Medical University, Chongqing, China; ^4^Department of Cardiology, Children’s Hospital of Chongqing Medical University, Chongqing, China; ^5^Department of Ultrasound, Chongqing General Hospital, Chongqing, China

**Keywords:** pulmonary arterial hypertension, prostanoids, endothelin receptor antagonists, phosphodiesterase 5 inhibitors, precision therapy

## Abstract

**Background:**

Although pulmonary arterial hypertension (PAH) is a fatal disease, specific drugs have been used to treat PAH. These drugs predominantly target these three pathobiological pathways: Endothelin receptor antagonist (ERA), nitric oxide (NO), and prostanoids pathways. In this review, we aimed to analyze the efficacy and safety of oral targeted treatments for PAH.

**Methods:**

The national library of medicine (MEDLINE), excerpta medica database (EMBASE), and Cochrane Central Register of Controlled Trials databases were searched. Randomized controlled trials that compared the oral targeted drugs with placebos were selected. We calculated odds ratios (ORs) with 95% confidence intervals (CIs) for variables with dichotomous outcomes, and standardized mean differences with continuous outcomes variables. Additionally, the mean of the differences for the 6-min walk distance (6MWD) was analyzed.

**Results:**

In total, 23 studies involving 7,121 patients were included in this study. These studies show that orally PAH-specific drugs could decrease the risk of clinical worsening events, with an OR of 0.55 (*p* < 0.001). Furthermore, these drugs could improve exercise capacity, showing a 21.74-m increase in 6MWD (95% CI: 17.53–25.95 m) and cause a greater amelioration of functional class (OR = 0.60, 95% CI: 0.47–0.76). Additionally, subgroup analysis indicated that compared with placebo, ERAs, and drugs in the NO pathway were most effective and safe, which are associated with an improvement in exercise capacity, 6MWD, and worsening events-free survival rate.

**Conclusion:**

Nitric oxide exhibited the most prominent clinical effect on exercise tolerance. However, in the subgroup analysis, oral targeted drugs of different pathways show applicability to different populations, which highlights the need for precise treatment in the clinical setting.

**Systematic Review Registration:**

[https://www.crd.york.ac.uk/prospero/display_record.php?RecordID=297946], identifier [CRD 42022297946].

## Background

Pulmonary arterial hypertension (PAH) is a fatal disease that exhibits pulmonary vascular remodeling. It is characterized by progressively increasing pulmonary artery pressure (PAP) and pulmonary vascular resistance (PVR). PAH eventually leads to hypoxia, right-sided heart failure, and death (1). According to guidelines, PAH is defined as a mean baseline PAP of less than 25 mm Hg, associated with pulmonary artery wedge pressure ≤15 mm Hg and elevated PVR ≥ 3 Wood units *via* right heart catheterization. PAH is generally classified into the following: (1) idiopathic or heritable; (2) drug or toxin-induced (aminorex, benfluorex, and toxic rapeseed oil); and (3) associated condition-induced, such as connective tissue disease, human immunodeficiency virus infection, congenital heart disease, and schistosomiasis (1). Since the 1990s, various drugs have been used to treat specific-PAH types, predominantly focusing on three pathobiological pathways: endothelin receptor antagonist (ERA), nitric oxide (NO), and prostanoids pathways (2), which significantly improve exercise capacity and cardiopulmonary parameters. These agents may be administered through the following: ingestion, inhalation, subcutaneous injection, and intravenous injection (2, 3). However, previous studies have indicated that intravenous injection can lead to embolism and thrombosis. Additionally, local pain with subcutaneous injection is a common occurrence (4–6). Although inhalation has proven to be effective and safe, only a few drugs have been recommended by the American Heart Association/European Society of Hypertension. Additionally, the inhaled drugs are often short-acting and require multiple daily doses (7). Comparatively, ingestion is commonly used and patients usually show good compliance. In 2016, a quantitative meta-analysis found that, compared to a placebo, oral drugs can ameliorate exercise intolerance, increase pulmonary vascular remodeling, and improve quality of life (8). However, sitaxsentan, an ERA that was eradicated from the market due to fatal hepatotoxicity in 2010 (9), and imatinib, a PAH medication terminated due to severe side effects (subdural hematoma), were included in the study. As a guideline for PAH, in 2018, it was proposed that the following targeted treatments be recommended: bosentan, ambrisentan, and macitentan in the ERA pathway; sidenafil, tadalafil, vardenafil, and udenafil in the NO pathway; and iloprost, beraprost, selexipag, and treprostinil in the prostanoids pathway (10). With studies [Fu et al. (11); etc.] indicating more proof about the efficacy and safety of targeted drugs of each type, including ingestion, inhalation, subcutaneous injection, and intravenous injection, focus has been put on the oral drugs as these are important treatments for PAH therapy. Early in 2018, Zheng et al. (12) meta-analyzed 25 randomized controlled trial (RCTs) about oral targeted therapies in PAH, showing the benefits of oral treatments on clinical worsening events (CWEs) in PAH. Accordingly, to provide the most suitable strategy for PAH populations with specific clinical characteristics for precision treatment, more detailed subgroup analyses are required. Hence, we conducted a meta-analysis and pooled data from clinical trials comparing oral PAH-specific drugs with placebo to assess the efficacy and safety of these treatments in different age groups and different functional classes (FCs).

## Methods

### Search strategy and article selection

This study complied with PRISMA (Preferred Reporting Items for Systematic Reviews and Meta-Analysis) guidelines. Two investigators (H-RZ and QL) independently searched PubMed, EMBASE, and Cochrane Central Register of Controlled Trials databases up to 10 December 2021. The full strategy is available in Table 1. Related manuscripts were manually searched to identify other eligible citations. The same authors independently screened the citations at the title and abstract level. Studies on the following were included: (1) randomized, placebo-controlled clinical trials; (2) patients diagnosed with PAH; (3) PAH-specific therapies with oral administration [oral prostanoids, ERAs, phosphodiesterase-5 inhibitor, prostacyclin receptor agonists, receptor tyrosine kinase inhibitors (RTK), and soluble guanylate cyclase stimulators]; and (4) one of the following outcomes: (a) safety outcomes (CWEs and adverse drug responses; b) exercise capacity outcomes (6-min walk distance, FC, and cardiopulmonary hemodynamics). These trials were then excluded if: (1) a combination therapy with other forms of drugs for patients with PAH was used; (2) the data included the use of a prohibited drug; (3) studies included persistent pulmonary hypertension in newborns; (4) acute hemodynamic responses to drugs occurred, and (5) sample size > 50. The full texts of the potentially eligible studies were then reviewed. Disagreements were resolved by a consensus with a third investigator (X-JJ). Patient and public involvement statements and ethical approval were not necessary for a secondary study.

**TABLE 1:** The detailed search strategy.

MEDLINE	

No.	Query
#1	controlled clinical trial [pt]
#2	randomized [tiab]
#3	randomly [tiab]
#4	clinical trials as topic [mesh: noexp]
#5	**#1 OR #2 OR #3 OR #4**
#6	hypertension, pulmonary [mh]
#7	pulmonary hypertension [tw]
#8	PH [tw]
#9	pulmonary arterial hypertension [tw]
#10	PAH [tw]
#11	idiopathic pulmonary arterial hypertension [tw]
#12	IPAH [tw]
#13	primary pulmonary hypertension [tw]
#14	PPH [tw]
#15	**#6 OR #7 OR #8 OR #9 OR #10 OR #11 OR #12 OR #13 OR #14**
#16	Prostaglandins [mh]
#17	phosphodiesterase 5 inhibitors [mh]
#18	Receptors, Endothelin [mh]
#19	Prostanoids [tw]
#20	Prostacyclins [tw]
#21	endothelin receptor antagonists [tw]
#22	ERA [tw]
#23	phosphodiesterase 5 inhibitors [tw]
#24	phosphodiesterase type 5 inhibitors [tw]
#25	PDE-5i [tw]
#26	PDE-5 inhibitors [tw]
#27	soluble guanylate cyclase stimulators [tw]
#28	tyrosine kinase inhibitors [tw]
#29	bosentan [tw]
#30	terbogrel [tw]
#31	beraprost [tw]
#32	sildenafil [tw]
#33	ambrisentan [tw]
#34	tadalafil [tw]
#35	vardenafil [tw]
#36	imatinib [tw]
#37	treprostinil [tw]
#38	macitentan [tw]
#39	riociguat [tw]
#40	selexipag [tw]
#41	**#16 OR #17 OR #18 OR #19 OR #20 OR #21 OR #22 OR #23 OR #24 OR #25 OR #26 OR #27 OR #28 OR #29 OR #30 OR #31 OR #32 OR #33 OR #34 OR #35 OR #36 OR #37 OR #38 OR #39 OR #40**
#42	placebos [mh]
#43	placebo [tiab]
#44	**#42 OR #43**
#45	**#5 AND #15 AND #41 AND #44**
#1	randomized controlled trial.pt.
#2	controlled clinical trial.pt.
#3	randomized.ab.
#4	clinical trials as topic.sh.
#5	randomly.ab.
#6	trial.ti.
#7	**1 OR 2 OR 3 OR 4 OR 5 OR 6**
#8	exp hypertension, pulmonary/
#9	pulmonary hypertension.tw.
#10	PH.tw.
#11	pulmonary arterial hypertension.tw.
#12	PAH.tw.
#13	idiopathic pulmonary arterial hypertension.tw.
#14	IPAH.tw.
#15	primary pulmonary hypertension.tw.
#16	PPH.tw.
#17	**8 OR 9 OR 10 OR 11 OR 12 OR 13 OR 14 OR 15 OR 16**
#18	exp Prostaglandins/
#19	exp phosphodiesterase 5 inhibitors/
#20	exp receptors, endothelin/
#21	prostanoids.tw.
#22	prostacyclins.tw.
#23	endothelin receptor antagonists.tw.
#24	ERA.tw.
#25	phosphodiesterase 5 inhibitors.tw.
#26	phosphodiesterase type 5 inhibitors.tw.
#27	PDE-5i.tw.
#28	PDE-5 inhibitors.tw.
#29	soluble guanylate cyclase stimulators.tw.
#30	tyrosine kinase inhibitors.tw.
#31	bosentan.tw.
#32	terbogrel.tw.
#33	beraprost.tw.
#34	sildenafil.tw.
#35	ambrisentan.tw.
#36	tadalafil.tw.
#37	vardenafil.tw.
#38	imatinib.tw.
#39	treprostinil.tw.
#40	macitentan.tw.
#41	riociguat.tw.
#42	selexipag.tw.
#43	**18 OR 19 OR 20 OR 21 OR 22 OR 23 OR 24 OR 25 OR 26 OR 27 OR 28 OR 29 OR 30 OR 31 OR 32 OR 33 OR 34 OR 35 OR 36 OR 37 OR 38 OR 39 OR 40 OR 41 OR 42**
#44	placebo.ab.
#45	**7 AND 17 AND 43 AND 44**
#1	randomized: ti,ab
#2	randomly: ti,ab
#3	trial [ti]
#4	**#1 OR #2 OR #3**
#5	MeSH descriptor hypertension, pulmonary explode all trees
#6	pulmonary hypertension: ti,ab,kw
#7	PH: ti,ab,kw
#8	pulmonary arterial hypertension: ti,ab,kw
#9	PAH: ti,ab,kw
#10	idiopathic pulmonary arterial hypertension: ti,ab,kw
#11	IPAH: ti,ab,kw
#12	primary pulmonary hypertension: ti,ab,kw
#13	PPH: ti,ab,kw
#14	**#5 OR #6 OR #7 OR #8 OR#9 OR #10 OR #11 OR #12 OR #13**
#15	MeSH descriptor Prostaglandins explode all trees
#16	MeSH descriptor phosphodiesterase 5 inhibitors explode all trees
#17	MeSH descriptor Receptors, Endothelin explode all trees
#18	Prostanoids: ti,ab,kw
#19	Prostacyclins: ti,ab,kw
#20	endothelin receptor antagonists: ti,ab,kw
#21	ERA: ti,ab,kw
#22	phosphodiesterase 5 inhibitors: ti,ab,kw
#23	phosphodiesterase type 5 inhibitors: ti,ab,kw
#24	PDE-5i: ti,ab,kw
#25	PDE-5 inhibitors: ti,ab,kw
#26	soluble guanylate cyclase stimulators: ti,ab,kw
#27	tyrosine kinase inhibitors: ti,ab,kw
#28	bosentan: ti,ab,kw
#29	terbogrel: ti,ab,kw
#30	beraprost: ti,ab,kw
#31	sildenafil: ti,ab,kw
#32	ambrisentan: ti,ab,kw
#33	tadalafil: ti,ab,kw
#34	vardenafil: ti,ab,kw
#35	imatinib: ti,ab,kw
#36	treprostinil: ti,ab,kw
#37	macitentan: ti,ab,kw
#38	riociguat: ti,ab,kw
#39	selexipag: ti,ab,kw
#40	**#15 OR #16 OR #17 OR #18 OR #19 OR #20 OR #21 OR #22 OR #23 OR #24 OR #25 OR #26 OR #27 OR #28 OR #29 OR #30 OR #31 OR #32 OR #33 OR #34 OR #35 OR #36 OR #37 OR #38 OR #39 OR #40**
#41	placebo: ti,ab
#42	**#4 AND #14 AND #40 #41**

### Data extraction and quality assessment

The following data were extracted from each article: first author, year of publication, duration of medication administration, number of participants in each group, treatment regimen, daily dosage, clinical characteristics of participants, CWEs, effects on a 6-min walk distance (6MWD), and hemodynamic values [mean pulmonary arterial pressure (mPAP), PVR, mean right atrial pressure, cardiac index, and pulmonary capillary wedge pressure (PCWP)]. CWEs were defined as all-cause mortality, lung or heart-lung transplantation, hospitalization for PAH, and escalation of treatment, which could be identified based on the original data documented from the articles or calculated from the events reported. The quality of the included trials was gaged according to a previous systematic review by using the Cochrane Collaboration’s risk-of-bias tool (13), including the following six domains: (1) allocation sequence generation, (2) allocation concealment, (3) blinding of participants, personnel, and outcome assessors, (4) completeness of participant follow-up, (5) handling of incomplete outcome data, and (6) protection against selective outcome reporting. Contact the author by email if necessary.

### Data synthesis and statistical analysis

Review Manager version 5.3 was used for statistical analysis. For all treatment comparisons, the random or fixed-effect model was applied according to the heterogeneity among studies, using the χ^2^ test the *I*^2^ statistic, with values > 50% (or *p* of χ^2^ test < 0.01), indicating substantial heterogeneity. Multi-arm studies were assessed by comparing each active arm with the control group separately. We calculated odds ratios (ORs) with 95% confidence intervals (CIs) for dichotomous outcomes, and calculated standardized mean differences (SMDs) for continuous outcomes, with mean of differences (MD) for 6MWD. To assess publication bias, we examined the funnel plot for evidence of small-study effects.

## Results

### Characteristics of the studies included in the meta-analysis

**FIGURE 1:**
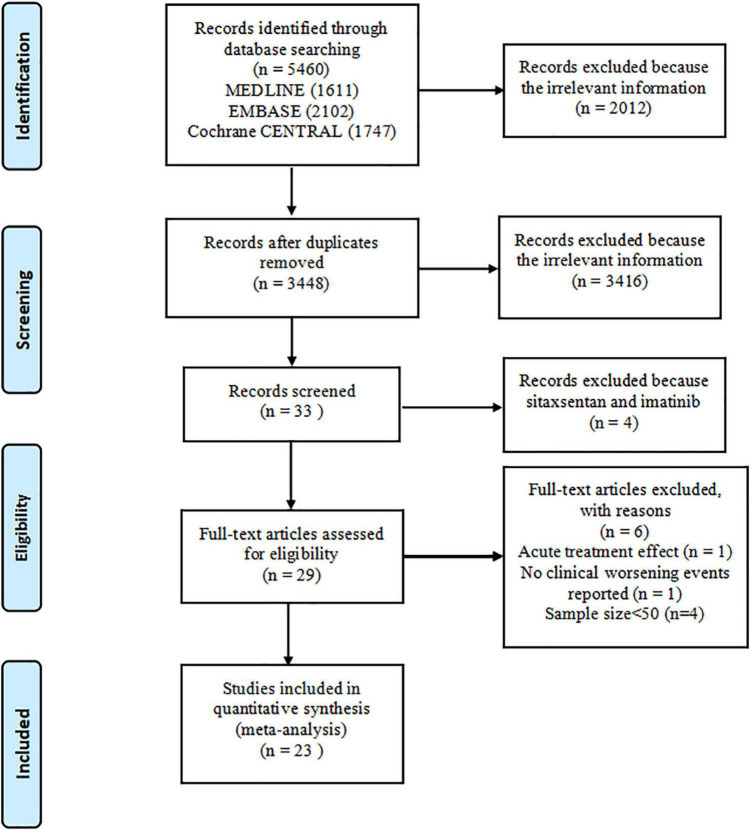
Flowchart.

Overall, 5,460 records were identified by literature search (PubMed 1,611 articles, EMBASE 2,102 articles, and the Cochrane Library 1,747 articles), and 3,448 records were recorded after 2,012 duplicates were removed. A total of 3,416 records were excluded after a review of titles and abstracts, which included duplications, reviews, case reports, animal studies, or studies irrelevant to our analysis. Additionally, four articles about sitaxsentan and imatinib were removed (14–17). The remaining 29 citations underwent detailed assessment at the full-text level, where one RCT evaluated the acute vascular effects (18), one trial did not record CWEs (19), and four trials did not meet sample size requirements (20–23). Eventually, 23 trials (24–46) meeting the inclusion criteria were pooled in this meta-analysis. The screening process is illustrated in Figure 1. These studies were conducted from 2002 to 2021 and encompassed 7,121 patients, most of which were multicenter studies. The treatment duration of oral targeted drugs in all studies ranged from 12 to 115 weeks, with a median treatment duration of 12 weeks. The FC of patients was evaluated at baseline and was mostly in II or III according to World Health Organization criteria, and details are shown in Table 2.

**TABLE 2:** Characteristics of the studies included in the meta-analysis.

Study	Age (y)	Period (s)	Treatment (*n*)	Control (*n*)	Treatment	Control	IPAH(%)	APAH(%)
**ERAs**								
Rubin et al. (24)	49	16	74	69	Bosentan (62.5–125 mg bid)	Placebo	73	27
Rubin et al. (24)	47	16	70	69	Bosentan (62.5–250 mg bid)	Placebo	67	33
Barst et al. (25)	51	18	60	62	Bosentan (125 mg bid)	Placebo	58	42
Galie et al. (26)	51	12	67	67	Ambrisentan (5 mg qd)	Placebo	63	37
Galie et al. (26)	48	12	68	67	Ambrisentan (10 mg qd)	Placebo	62	38
Galie et al. (26)	52	12	64	65	Ambrisentan (2.5 mg qd)	Placebo	65	35
Galie et al. (26)	51	12	63	65	Ambrisentan (5 mg qd)	Placebo	65	35
Galie et al. (27)	45	26	93	92	Bosentan (62.5–125 mg bid)	Placebo	61	39
Pulido et al. (28)	46	115	250	250	Macitentan (3 mg qd)	Placebo	54	43
Pulido et al. (28)	46	115	242	250	Macitentan (10 mg qd)	Placebo	53	46
Mclaughlin et al. (29)	54	16	159	175	Bosentan (max125 mg bid)	Placebo	64	35
**NO**								
Galie et al. (30)	48	12	69	70	Sidenafil (20 mg tid)	Placebo	62	38
Galie et al. (30)	50	12	67	70	Sidenafil (40 mg tid)	Placebo	62	38
Galie et al. (30)	49	12	71	70	Sidenafil (80 mg tid)	Placebo	62	38
Galie et al. (31)	55	16	82	82	Tadalafil (2.5 mg qd)	Placebo	–	40
Galie et al. (31)	55	16	80	82	Tadalafil (10 mg qd)	Placebo	–	35
Galie et al. (31)	54	16	82	82	Tadalafil (20 mg qd)	Placebo	–	37
Galie et al. (31)	54	16	79	82	Tadalafil (40 mg qd)	Placebo	–	38
Barst et al. (32)	51	16	45	45	Tadalafil (20 mg qd)	Placebo	–	38
Barst et al. (32)	51	16	42	45	Tadalafil (40 mg qd)	Placebo	–	39
Barst et al. (32)	58	16	37	37	Tadalafil (20 mg qd)	Placebo	–	35
Barst et al. (32)	57	16	37	37	Tadalafil (40 mg qd)	Placebo	–	36
Jing et al. (33)	31	12	44	20	Vardenafil (5 mg bid)	Placebo	59	41
Ghofrani et al. (34)	50	12	63	126	Riociguat (max1.5 mg tid)	Placebo	65	34
Ghofrani et al. (34)	51	12	254	126	Riociguat (max2.5 mg tid)	Placebo	61	37
Zhuang et al. (35)	52	16	60	64	Tadalafil (40 mg qd)	Placebo	–	37
Vizza et al. (36)	56	12	50	53	Sidenafil (20 mg tid)	Placebo	–	35
Chang et al. (37)	47	16	31	32	Udenafil (50 mg qd)	Placebo	51	49
**Prostanoids**								
Galie et al. (38)	45	12	65	65	Beraprost (max0.12 mg qid)	Placebo	48	52
Barst et al. (39)	42	52	60	56	Beraprost (max0.2 mg qid)	Placebo	74	26
Tapson et al. (40)	51	16	174	176	Treprostinil (0.5–16 mg bid)	Placebo	–	34
Tapson et al. (41)	51	16	157	153	Treprostinil (min0.25 mg bid)	Placebo	–	35
Jing et al. (42)	42	12	233	116	Treprostinil (min0.125 mg bid)	Placebo	–	26
Sitbon et al. (43)	48	26	574	582	Selexipag (0.2–1.6 mg bid)	Placebo	56	42
Torres et al. (44)	49	22	40	21	Ralinepag (0.01–0.3 mg bid)	Placebo	53	38
White et al. (45)	45	24	346	344	Treprostinil (max12 mg tid)	Placebo	–	37
Chin et al. (46)	52	26	123	124	Selexipag (0.2–1.6 mg bid)	Placebo	47	47

### Safety

Data on the safety of the overall oral targeted treatment for PAH were obtained from 23 articles, where no heterogeneity existed (*I*^2^ = 0.0%). The fixed effects model showed that oral targeted therapy could improve mortality compared with the control group (OR = 0.55, 95% CI, range: 0.49–0.62, *p* < 0.001). Furthermore, three approaches lowered the occurrence of CWEs: with OR = 0.49 (95% CI, range: 0.37–0.66, *p* < 0.001) in ERA, OR = 0.46 (95% CI, range: 0.34–0.62, *p* < 0.001) in NO, and OR = 0.58 (95% CI, range: 0.49–0.69, *p* < 0.001) in prostanoids. The frequency of adverse drug responses to these oral agents for PAH was detected in 23 citations, and the three most common adverse event (AEs) were headache (37.3%, 95% CI, range: 36.1–38.5%), diarrhea (19.9%, 95% CI, range: 18.7–21.1%), and nausea (20.1%, 95% CI, range: 18.8–21.4%). Subgroup analysis showed that the three most common AEs in ERA were headache (12.5%, 95% CI, range: 10.7–14.4%), peripheral edema (13.9%, 95% CI, range: 11.7–16.0%), and upper respiratory tract infection (16.4%, 95% CI, range: 13.6–19.3%); headache (31.4%, 95% CI, range: 28.9–34.0%), diarrhea (9.8%, 95% CI, range: 8.1–11.5%), and dyspepsia (8.6%, 95% CI, range: 6.9–10.2%) in NO; and headache (72.1%, 95% CI, range: 70.1–74.2%), diarrhea (49.6%, 95% CI, range: 47.4–51.9%), and nausea (40.3%, 95% CI, range: 38.1–42.5%) in prostanoids.

### Exercise capacity

**FIGURE 2:**
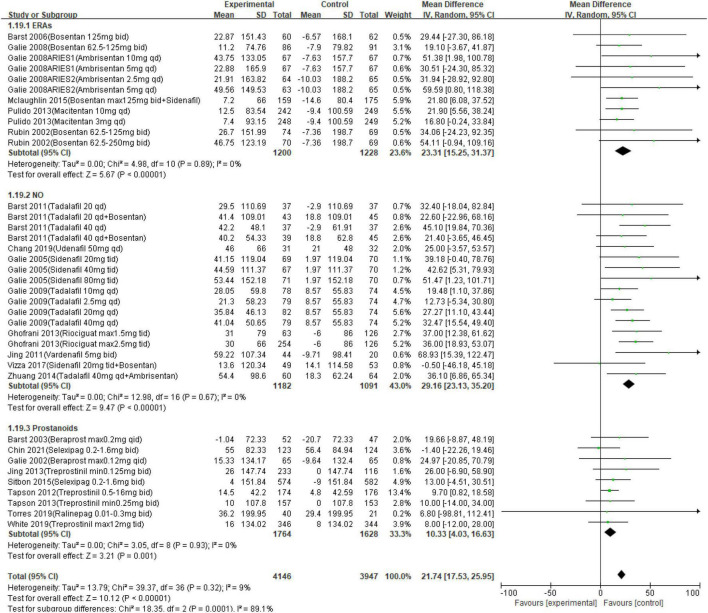
Meta-analysis of 6-min walk test (6MWD) in different pathways.

Twenty-seven studies reported a change in 6MWD from baseline to the endpoints (a short-to medium-term treatment of no more than 2 years; Table 3). Outcomes of meta-analysis in 6MWD demonstrated that PAH-specific drugs in oral administration improve 6MWD by 21.74 m (95% CI from 17.53 to 25.95 m, *p* < 0.001). Consequently, in different approaches, compared with placebo, oral drugs in ERA elevated 6MWD by 23.31 m (95% CI, range: 15.25–31.37, *p* < 0.001; *I*^2^ = 0.0%), and a 29.16-m improvement in 6MWD was detected in the NO way (95% CI, range: 23.13–35.20, *p* < 0.001; *I*^2^ = 0.0%), of 10.33 m in the prostanoids way (95% CI, range: 4.03–16.63, *p* < 0.001, *I*^2^ = 0.0%). The details are presented in Figure 2. Changes in FC were detected in each citation, showing improved or remained state in 92.0% of enrolled patients (95% CI, range: 91.0–93.1%), and only worsened in 8.0% (95% CI, range: 6.9–9.0%). Hence, the data showed that a short-medium term of oral PAH-specific medication administration was statistically associated with an obvious improvement in FC (OR = 0.60, 95% CI, range: 0.47–0.76, *p* < 0.001; *I*^2^ = 31%), where an apparent amelioration was only found in drugs when targeting prostanoids (*p* = 0.04) and NO (*p* < 0.001), while no significant change targeting ERAs (*p* = 0.12).

**TABLE 3:** Data on safety, exercise capacity, and hemodynamics for different drugs.

Types of drugs	Number of articles	Pooling models	Heterogeneity *I*^2^ (%)	Effect estimate (95%CI)	*p*
**ERA**					
Worsening events	6	Random effects model	37	OR: 0.49 (0.37, 0.66)	<0.001
6MWD	6	Fixed effects model	0	MD: 23.31 (15.25, 31.37)	<0.001
FC	4	Random effects model	32	OR: 0.56 (0.27, 1.15)	0.12
mPAP	0	–	–	–	–
PVR	0	–	–	–	–
mRAP	0	–	–	–	–
CI	2	Fixed effects model	0	SMD: 0.40 (0.20, 0.61)	<0.001
PCWP	0	–	–	–	–
**NO**					
Worsening events	8	Fixed effects model	0	OR: 0.46 (0.34, 0.62)	<0.001
6MWD	8	Fixed effects model	0	MD: 29.16 (23.13, 35.20)	<0.001
FC	6	Random effects model	5	OR: 0.53 (0.38, 0.74)	<0.001
mPAP	4	Random effects model	34	SMD: −0.43 (−0.59, −0.26)	<0.001
PVR	3	Random effects model	0	SMD: −0.63 (−0.76, −0.49)	<0.001
mRAP	3	Fixed effects model	0	SMD: −0.28 (−0.41, −0.15)	<0.001
CI	3	Fixed effects model	0	SMD: 0.42 (0.24, 0.60)	<0.001
PCWP	2	Fixed effects model	0	SMD: 0.11 (−0.07, 0.28)	0.23
**Prostanoids**					
Worsening events	9	Random effects model	0	OR: 0.58 (0.49, 0.69)	<0.001
6MWD	9	Fixed effects model	0	MD: 10.33 (4.03, 16.63)	<0.001
FC	8	Random effects model	53	OR: 0.65 (0.43, 0.98)	0.04
mPAP	4	Random effects model	88	SMD: −0.51 (−1.08, 0.06)	0.08
PVR	1	–	–	SMD: −0.64 (−1.18, −0.10)	0.02
mRAP	4	Random effects model	94	SMD: 0.62 (−0.49, 1.74)	0.27
CI	4	Random effects model	90	SMD: 0.60 (−0.06, 1.26)	0.07
PCWP	1	–	–	SMD: −0.15 (−0.71, 0.41)	0.60

### Correlation between worsening events and exercise capacity

**FIGURE 3:**
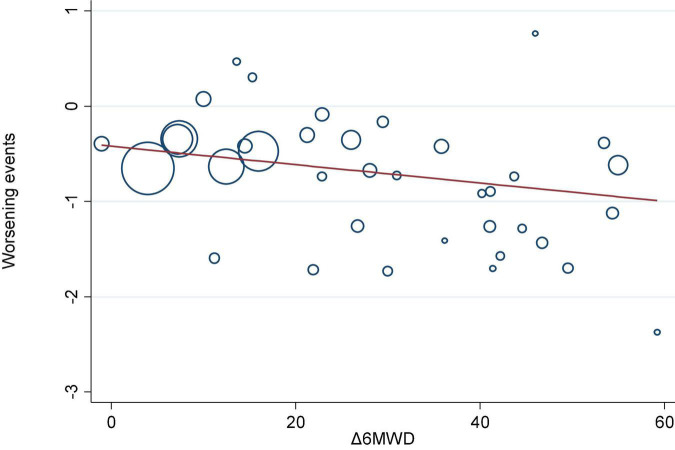
Meta-regression analysis of 6-min walk distance from baseline (Δ6MWD) and clinical worsening events (CWEs).

**FIGURE 4:**
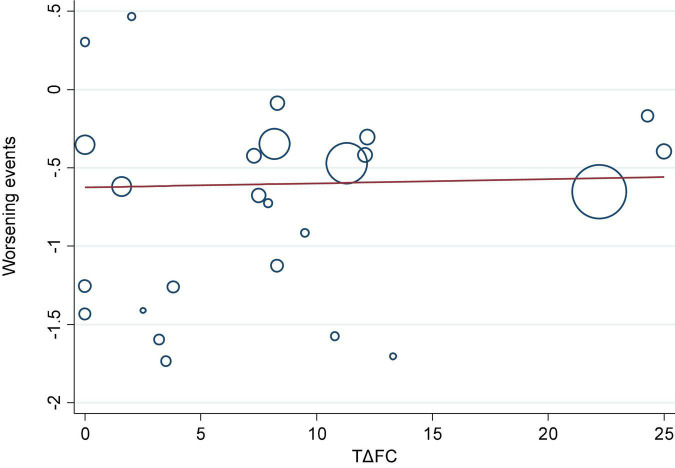
Meta-regression analysis of change in functional class from baseline (ΔFC) and clinical worsening events (CWEs).

We investigated the correlation between AEs and exercise tolerance in participants treated with active drugs. In the meta-regression analysis, we found few relationships between the change in 6MWD from baseline (Δ6MWD) and CWEs (*p* = 0.032; Figure 3). We also found no relationship between CWEs and deterioration rate of FC (*p* = 0.775; Figure 4).

### Cardiopulmonary hemodynamic parameters

Cardiopulmonary hemodynamics have been achieved in some studies (27, 28, 30, 33, 34, 37–39, 44, 46). With regard to mPAP, 8 articles (30, 33, 34, 37–39, 44, 46) have shown a decrease compared to that of oral targeted drugs with placebo (SMD = −0.42, 95% CI, range: −0.63 to −0.21, *p* < 0.001; *I*^2^ = 72.0%), and the changes were associated with a similar trend in PVR (SMD = −0.63, 95% CI, range: −0.76 to −0.50, *p* < 0.001; *I*^2^ = 0.0%) and mRAP (SMD = −0.06, 95% CI, range: −0.35 to −0.23, *p* = 0.67; *I*^2^ = 83.0%), but there were rare improvements in PCWP (SMD = 0.09, 95% CI, range: −0.08–0.25, *p* = 0.32; *I*^2^ = 0.0%). For systemic circulation, oral drugs targeting PAH could improve CI with an SMD of 0.45 (95% CI, range: 0.25–0.66, *p* < 0.001; *I*^2^ = 70.0%). The details of the different approaches are shown in Table 4.

**TABLE 4:** Data on safety, exercise capacity, and hemodynamics from included studies.

Parameters of article	Number of articles	Pooling models	Heterogeneity *I*^2^ (%)	Effect estimate (95%CI)	*p*
**Safety**					
Worsening events	23	Fixed effects model	0	OR: 0.55 (0.49, 0.62)	<0.001
**Exercise capacity**					
6MWD	23	Random effects model	9	MD: 21.74 (17.53, 25.95)	<0.001
FC	17	Random effects model	31	OR: 0.60 (0.47, 0.76)	<0.001
**Hemodynamics**					
mPAP	8	Random effects model	72	SMD: −0.42 (−0.63, −0.21)	<0.001
PVR	4	Fixed effects model	0	SMD: −0.63 (−0.76, −0.50)	<0.001
mRAP	7	Random effects model	83	SMD: −0.06 (−0.35, −0.23)	0.67
CI	9	Random effects model	70	SMD: 0.45 (0.25, 0.66)	<0.001
PCWP	3	Fixed effects model	0	SMD: 0.09 (−0.08, 0.25)	0.32

### Subgroup analysis

#### Functional class-treatment subgroup analyses

Participants in the study were classified as grades I–II or III–IV based on their initial FC ratings. We performed subgroup analysis based on the FC grade of the treatment group.

As shown in Table 5, patients with FC I–II improved by 21.60 m after oral treatment (95% CI, range: 12.86–30.34, *p* < 0.001), but only ERAs (MD: 19.74, 95% CI, range: 9.42–30.06, *p* < 0.001) increased among the three classical pathway targeted drugs. In patients with FC III–IV, oral targeted drugs of three classical pathways, ERAs, NO, and prostanoids, all showed improvement at 6MWD of 28.88 m (95% CI: 15.99–41.78), 27.38 m (95% CI, range: 21.08–33.69), and 10.08 m (95% CI, range: 3.24–16.92), respectively.

**TABLE 5:** Subgroup meta-analysis based on age and functional class (FC) grade.

Groups	Types of drugs	Number of articles	6MWD			Worsening events		
			MD	95%CI	*P*	OR	95%CI	*P*
**Age**								
<50		13	22.80	15.52–30.09	<0.001	0.55	0.48–0.64	<0.001
	ERAs	4	22.23	12.30–32.15	<0.001	0.48	0.34–0.69	<0.001
	Prostanoids	6	17.08	4.25–29.92	0.009	0.57	0.47–0.68	<0.001
	NO	3	38.37	18.79–57.95	<0.001	0.50	0.20–1.27	0.14
≥50		12	22.96	16.82–29.10	<0.001	0.54	0.43–0.68	<0.001
	ERAs	3	25.41	11.61–39.21	<0.001	0.47	0.25–0.88	0.02
	Prostanoids	3	8.20	0.47–15.94	0.04	0.68	0.43–1.08	0.11
	NO	6	28.20	21.85–34.54	<0.001	0.45	0.32–0.63	<0.001
**FC**								
I–II		7	21.60	12.86–30.34	<0.001	0.50	0.36–0.70	<0.001
	ERAs	3	19.74	9.42–30.06	<0.001	0.48	0.30–0.77	0.002
	Prostanoids	2	18.79	−8.76–46.33	0.18	0.50	0.21–1.15	0.10
	NO	2	30.42	9.99–50.86	0.004	0.60	0.11–3.36	0.56
III–IV		16	22.23	16.98–27.48	<0.001	0.53	0.46–0.62	<0.001
	ERAs	4	28.88	15.99–41.78	<0.001	0.47	0.30–0.74	0.001
	Prostanoids	6	10.08	3.24–16.92	0.004	0.57	0.47–0.70	<0.001
	NO	6	27.38	21.08–33.69	<0.001	0.46	0.33–0.64	<0.001

The OR estimate for CWEs was a reduction of 50% (95% CI, range: 0.36–0.70, *p* < 0.001) in FC I–II; only ERAs reduced the OR of CWEs by 0.52 (*p* = 0.002), while NO and prostanoids decreased by 0.40 and 0.50, respectively, which was not statistically significant. However, the CWES results of oral treatment of FC III-IV patients showed the same trend as that of the 6MWD. The ORs of ERA, NO, and prostanoids were 0.47, 0.46, and 0.57, respectively.

#### Age-treatment subgroup analyses

As shown in Table 5, we divided the treatment group into two groups (<50 years and ≥50 years) according to the age of patients to observe the therapeutic effect of the different types of drugs in various age stages.

In younger populations (<50 years), agents of the NO pathway reduced the risk of CWEs, with OR = 0.50 (95% CI, range: 0.20–1.27, *p* = 0.14; *I*^2^ = 21.0%), and an OR of 0.48 in ERAs (95% CI, range: 0.34–0.69, *p* < 0.001; *I*^2^ = 35.0%), whereas prostanoids reduced the OR of CWEs by 43% (95% CI, range: 0.47–0.68, *p* < 0.001; *I*^2^ = 0.0%). ERAs lowered the risk of CWEs, with an OR of 0.47 (95% CI, range: 0.25–0.88, *p* = 0.02; *I*^2^ = 50.0%), associated with an OR of 0.45 in NO (95% CI, range: 0.32–0.63, *p* < 0.001; *I*^2^ = 0.0%) and 0.68 in prostanoids (*p* = 0.11) in the older population (≥50 years).

As shown in 6MWD, NO increased by 38.37 m in 6MWD (95% CI, range: 18.79–57.95 m, *p* < 0.001; *I*^2^ = 0.0%), and ERAs and prostanoids also improved by 22.23 m (95% CI, range: 12.30–32.15 m, *p* < 0.001; *I*^2^ = 0.0%) and 17.08 m of 6MWD (95% CI, range: 4.25–29.92 m, *p* = 0.009; *I*^2^ = 0.0%), respectively, in younger patients (<50 years). In the older populations, NO increased 6MWD by 28.20 m (95% CI, range: 21.85–34.54, *p* < 0.001; *I*^2^ = 0.0%), and a 25.41-m elevation of 6MWD was detected in ERAs (95% CI, range: 11.61–39.21, *p* < 0.001; *I*^2^ = 0.0%), with 8.20-m elevation in prostanoids (95% CI, range: 0.47, 15.94, *p* = 0.04; *I*^2^ = 0.0%).

As demonstrated above, PAH-targeted drugs could significantly reduce the incidence of worsening events in ERAs, NO, and prostanoids. In the condition, subgroup analysis further indicated that when compared to placebo, macitentan lowered the risk of worsening events, with an OR of 0.61 (*p* < 0.001), associated with an OR of 0.54 in bosentan (*p* < 0.001) and 0.27 in ambrisentan (*p* < 0.001). In the NO pathway, tadalafil, riociguat, and vardenafil contributed to a decreased risk of CWEs, with an OR of 0.47 (*p* < 0.001), 0.28 (*p* = 0.02), and 0.09 (*p* = 0.04), respectively, but a non-obvious outcome was found in patients treated with sildenafil and udenafil. Furthermore, in prostanoids pathway, only treprostinil with oral administration and selexipag were detected to lead to a low risk of mortality, with an OR of 0.67 and 0.52, respectively.

As data presented in 6MWD, subgroup analysis demonstrated an obvious improvement when patients were treated with ERAs, where bosentan increased 6MWD by 23.46 m (95% CI, range: 11.42–35.49, *p* < 0.001), and 43.84-m elevation of 6MWD were detected in ambrisentan, with 19.46-m elevation in macitentan (95% CI, range: 7.66–31.25, *p* = 0.001). In the NO pathway, except for udenafil, other drugs (tadalafil, sidenafil, riociguat, and vardenafil) detected an obvious elevation of 6MWD, and an opposite trend was found in prostanoids; only treprostinil showed an improvement in 6MWD (MD = 10.34 m, 95% CI, range: 2.85–17.82 m, *p* = 0.007; details in Table 6).

**TABLE 6:** Subgroup meta-analysis based on drug classes and drugs.

Drugs	Numbers of articles	6MWD			Worsening events		
		MD	95%CI	*P*	OR	95%CI	P
**ERA**							
Bosentan	4	23.46	11.42–35.49	<0.001	0.54	0.38–0.75	<0.001
Ambrisentan	1	43.84	16.12–71.55	0.002	0.27	0.14–0.53	<0.001
Macitentan	1	19.46	7.66–31.25	0.001	0.61	0.46–0.82	<0.001
**NO**							
Tadalafil	3	26.15	18.81–33.50	<0.001	0.47	0.33–0.69	<0.001
Udenafil	1	25.00	−3.57–53.57	0.09	2.14	0.18–24.86	0.54
Sidenafil	2	33.95	12.79–55.12	0.002	0.55	0.27–1.11	0.10
Riociguat	1	36.32	22.30–50.35	<0.001	0.28	0.10–0.78	0.02
Vardenafil	1	68.93	15.39–122.47	0.01	0.09	0.01–0.90	0.04
**Prostanoids**							
Beraprost	2	21.14	−3.08–45.36	0.09	0.69	0.32–1.49	0.35
Treprostinil	4	10.34	2.85–17.82	0.007	0.67	0.51–0.88	0.003
Selexipag	2	6.96	−6.96–20.89	0.33	0.52	0.41–0.66	<0.001
Ralinepag	1	6.80	−98.99–112.41	0.90	0.24	0.02–2.86	0.26

#### Publication bias and quality assessment

**FIGURE 5:**
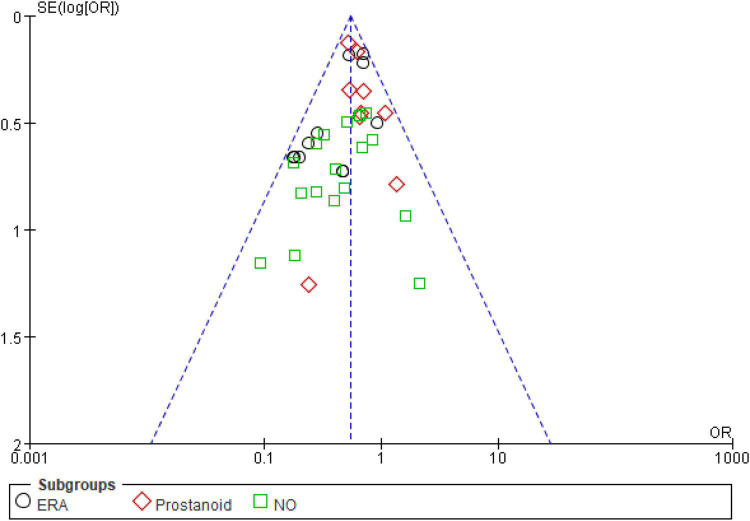
Funnel plot for the effect of oral targeted drugs on worsening events.

**FIGURE 6:**
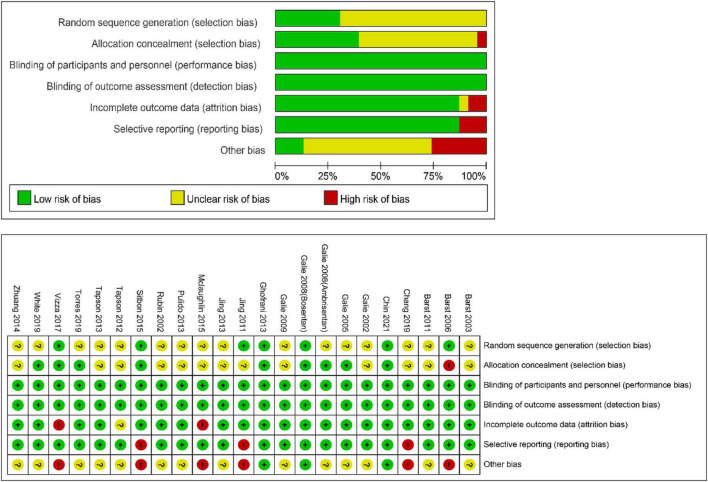
Cochrane collaboration’s risk-of-bias tool and risk of bias graph for the pooled studies in this meta-analysis.

For the meta-analysis of all oral targeted drugs on worsening events and 6MWD (Figure 5), there was no evidence of publication bias by inspection of the funnel plot. The details of the quality assessment of all pooled studies are shown in Figure 6.

## Discussion

Pulmonary arterial hypertension is a life-threatening disorder, and the 5-year survival rate is less than 60% (47). An approved drug could lengthen the lifespan of patients, but it cannot reverse the development of the disease. Targeted drugs, such as those that affect ERA, NO, and prostanoids pathways, are still the recommended therapy for PAH patients (2). As such, they have been shown to slow PAH progression and improve exercise capacity and cardiopulmonary hemodynamics. Furthermore, oral administration may be the common preference for patients with FC I–II. Although some drugs have yielded side effects (48), the symptoms are often mild and eventually clear without treatment. In 2015, the outcomes of a meta-analysis demonstrated that the oral administration of PAH-specific drugs is a safe and effective treatment. However, this study incorporated sitaxsentan and imatinib, which are no longer in clinical use. Meanwhile, there is no detailed description of the precise treatment of PAH patients of different ages and FC grades with oral drugs.

Prostanoids, ERAs, and NO signaling pathway drugs are all used to achieve therapeutic goals through vasodilation, while RTK drugs can theoretically treat PAH by inhibiting the pathogenesis pathway of PAH, but their drug imatinib is not approved for the treatment of PAH due to side effects, such as subdural hemorrhage. Therefore, the three major classical pathway drugs are still mainly used clinically to treat PAH, and these traditional methods still have a high mortality rate, although they alleviate the symptoms to some extent. Nanomedicines and gene therapy are emerging approaches for the treatment of PAH in the future, but a higher level of evidence is needed to reach the goal of individualized treatment (49). In the meta-regression analysis, there is a correlation between CWEs and the change in 6MWD (*p* = 0.032; Figure 3), which possibly emphasizes the need to monitor CWEs.

Most NO drugs (including tadalafil, vardenafil, and riociguat) were identified to be effective. Indeed, Galie et al. (30), Vizza et al. (36), and Chang et al. (37) have found that oral udenafil and sildenafil might not improve the exercise capacity or pulmonary vascular remodeling in short-term treatment. Even treprostinil, a type of prostanoids, was detected as the only agent with a significant amelioration in exercise capacity, where it showed an obvious change in hemodynamics of prostanoids. AEs reported in the three classical methods were mild to moderate, with a high prevalence of headache. Additionally, the most common severe adverse drug response in oral ERAs was right-sided heart failure, accounting for approximately 14.0% (95% CI, range: 10.9–17.1%), dyspnea in the NO pathway (approximately 5.7%, 95% CI, range: 4.2–7.2%), and prostanoids (16.0%, 95% CI, range: 13.3–18.6%), respectively.

Age-treatment subgroup analysis indicated that the oral administration of NO pathway agents improved exercise capacity, cardiopulmonary hemodynamics, and worsening events-free survival rate across all age groups (<50 years and ≥50 years). In the younger populations (<50 years), NO revealed a significant improvement in 6MWD (MD = 38.37, *p* < 0.001) and a reduction of 0.50 in the OR of CWEs (*p* = 0.14). Additionally, the data of NO demonstrated excellent clinical efficacy in the older patients, increasing 6MWD by 28.20 m (*p* < 0.001), and significantly reducing CWEs, with an OR of 0.45 (*p* < 0.001). Age-related vasculature alteration is an obvious risk factor for PAH (50). Furthermore, clinical registries (51) have mentioned age-related differences in functional status and hemodynamics in PAH where the mean age of patients was reported as 50–65 years old. The Scottish composite index constructed a multivariate Cox model based on data from 182 PAH patients, using age, sex, etiology, right atrial pressure, cardiac output, and 6MWD to predict survival (52). Yi et al. found that older patients (≥50 years) were more likely to die than younger patients (<50 years) based on PAH registries in the United Kingdom and Ireland (48). This means that age is a predominant factor affecting patient prognosis. ERAs and NO significantly reduced the OR of CWEs by 53% (*p* = 0.02) and 54% (*p* < 0.001), respectively. ERAs showed the greatest efficacy on CWEs and could be used as the first targeted oral agent in elderly patients (≥50 years). Additionally, PAH is mainly a disease in women, according to data from registries (53). The study did not perform a subgroup analysis of sex and PAH classification because most of the 23 studies included women, and most of the patients had primary PAH.

In the subgroup analysis, subdivided by FC, ERAs demonstrated superior clinical efficacy in patients with FC I–II, 6MWD increasing by 19.74 m and reducing the OR of CWEs by 52%. ERAs also showed the best clinical efficacy compared to that of placebo in patients with FC III–IV, with a 6MWD 28.88-m improvement and a 53% reduction in the OR of CWEs. Although FC was not included in the Cox model mentioned above, FC and age were also included in the prognostic prediction as relevant parameters in some prediction equations or models proposed (54). By subgroup analysis of age and FC, patients were stratified using evidence-based medicine to aid doctors in choosing more appropriate drugs to improve patient outcomes.

Currently, patients with pulmonary hypertension are clinically classified into the following five groups: (1) those with predominantly pulmonary artery disease; (2) those consisting of pulmonary hypertension associated with left heart disease; (3) those with PAH associated with pulmonary disease and hypoxia; (4) those with PAH due to chronic thromboembolism; and (5) those due to unclear multifactorial mechanisms. This classification is widely accepted and used in clinical practice, but this approach is not ideal for some patients. Precision therapy, which can also be referred to as stratified therapy, intervenes based on the greatest benefit and least harm for most patients (55). We build on this by again subgrouping patients using age and FC class, narrowing the patient population so that the majority of patients in the group are susceptible to the drugs used, and improving prognosis. The NO drugs showed excellent efficacy in all age groups, but not in patients with FC I–II. However, in the subgroup analysis of FC, ERAs showed the best clinical efficacy. In clinical practice, doctors can choose the corresponding treatment plan based on basic patient information. First, we determine the age of the patient and select the appropriate drug. Then, consider the FC grade of the patient and decide whether to add other drugs to form a dual or triple treatment. In general, the clinical conditions of patients taking oral drugs are not serious, which is why most of the patients involved in this study were in FC II–III. When the patient does not fall under any of these conditions, other treatments can be used, such as inhaled or intravenous drugs. When the level of individualized treatment is not reached, age and FC grade can be first subdivided into a small number of patients to achieve accurate treatment for this small population.

The limitations of this study are as follows: (1) the sample size of some studies was too small and there was great uncertainty; (2) only studies in English were included in this meta-analysis, and studies not included in other languages may result in partial data omission; (3) since this was a secondary study, it was difficult to obtain individual patient data, which may cause uncertainty in the data; (4) only some studies have reported hemodynamic changes; and (5) fewer studies were pooled in the subgroup analysis.

## Conclusion

In this meta-analysis, we assessed the efficacy and safety of orally targeted drugs for PAH. This study compared the clinical efficacy of three classical oral targeted drugs through subgroup analysis of age and FC grade, providing evidence-based medicine for precise clinical treatment.

## Data availability statement

The original contributions presented in this study are included in the article/Supplementary material, further inquiries can be directed to the corresponding author.

## Author contributions

H-RZ and QL collected the data. H-RZ and H-YK analyzed the data. H-RZ wrote the manuscript. H-YK modified the manuscript. X-JJ provided the idea. All authors contributed to the article and approved the submitted version.
